# BP–ANN Model Coupled with Particle Swarm Optimization for the Efficient Prediction of 2-Chlorophenol Removal in an Electro-Oxidation System

**DOI:** 10.3390/ijerph16142454

**Published:** 2019-07-10

**Authors:** Yu Mei, Jiaqian Yang, Yin Lu, Feilin Hao, Dongmei Xu, Hua Pan, Jiade Wang

**Affiliations:** 1 College of Environment, Zhejiang University of Technology, Hangzhou 310032, China; 2 College of Biological and Environmental Engineering, Zhejiang Shuren University, Hangzhou 310005, China

**Keywords:** BP–ANN, PSO–ANN, electro-oxidation

## Abstract

Electro-oxidation is an effective approach for the removal of 2-chlorophenol from wastewater. The modeling of the electrochemical process plays an important role in improving the efficiency of electrochemical treatment and increasing our understanding of electrochemical treatment without increasing the cost. The backpropagation artificial neural network (BP–ANN) model was applied to predict chemical oxygen demand (COD) removal efficiency and total energy consumption (TEC). Current density, pH, supporting electrolyte concentration, and oxidation–reduction potential (ORP) were used as input parameters in the 2-chlorophenol synthetic wastewater model. Prediction accuracy was increased by using particle swarm optimization coupled with BP–ANN to optimize weight and threshold values. The particle swarm optimization BP–ANN (PSO–BP–ANN) for the efficient prediction of COD removal efficiency and TEC for testing data showed high correlation coefficient of 0.99 and 0.9944 and a mean square error of 0.0015526 and 0.0023456. The weight matrix analysis indicated that the correlation of the five input parameters was a current density of 18.85%, an initial pH 21.11%, an electrolyte concentration 19.69%, an oxidation time of 21.30%, and an ORP of 19.05%. The analysis of removal kinetics indicated that oxidation–reduction potential (ORP) is closely correlated with the chemical oxygen demand (COD) and total energy consumption (TEC) of the electro-oxidation degradation of 2-chlorophenol in wastewater.

## 1. Introduction

Wastewater produced by various industrial processes contains large quantities of chlorophenol compounds, which are highly toxic and resistant to biological degradation [1]. The compound 2-chlorophenol is a typical chlorophenol compound that is listed as a priority pollutant by the Environmental Protection Agency, given its carcinogenic properties [2,3]. Electro-oxidation, an effective technology that does not require the use of extra reagents, is commonly used to remove chlorophenol compounds from wastewater because of its high efficiency, rapid reaction rate, and environmental friendliness [4,5]. However, the energy cost of the electro-oxidation process limits its application [6].

The establishment of appropriate models for electro-oxidation is essential given the complexity of this process. Modeling of the electrochemical process plays an important role in improving the efficiency of electrochemical treatment and a further understanding of electrochemical treatment without increasing the cost. Empirical models and semi-empirical models, such as pseudo-first-order kinetics [7], pseudo-second-order kinetics [8], a computational fluid dynamics (CFD) model, and response surface methodology (RSM) model, are usually established for the prediction of electrochemical process behaviors. Bu et al. [9] established the kinetic model of the degradation of oxcarbazepine (OXC) using electrochemically-activated persulfate (EC/PS) based on two assumptions. Conventional mathematical or mechanistic models can be used to predict the final state of the system only under given circumstances [10]. Wang et al. [11] calculated the velocity distribution and turbulence distribution of a new type of tubular plunger flow reactor by CFD. CFD can reveal the mass transfer process and mechanism of an electrochemical reactor, but it is still affected by grid mass, transfer mode, and calculation [12,13]. Song et al. [14] optimized the electrochemical simultaneous removal of the ammonia nitrogen process using RSM, which showed a good prediction. The main disadvantage of RSM is that it cannot effectively improve approximation accuracy, even with an increase in the number of sample points. Electrochemistry is a complex non-linear process, and it is difficult to explain it clearly through traditional empirical and semi-empirical modeling.

In contrast to traditional mathematical models, scholars have done some research on the non-linear prediction model of the electrochemical process. Artificial Neural Networks (ANNs) do not require the modeling of a detailed mathematical formulation of a system and have been used to determine complex relationships between input and output data [15]. Daneshvar et al. [16] established an ANN model for the decolorization process of dyeing wastewater by electroflocculation. This model can predict the color removal rate under different experimental conditions. Researchers pointed out that ANN has good prospects for the prediction of complex systems [17,18]. Belkacem et al. [19] applied a backpropagation artificial neural network (BP–ANN) prediction of oxytetracycline removal in an electro-oxidation system, which chose 14 nodes from the hidden layer, the LM (Levenberg-Marquardt)algorithm, the logsig transfer function of the hidden layer, and the purelin transfer function of the output layer. However, the researchers did not verify the reliability of the network or compare the algorithms and transfer functions on the network. Moreover, BP–ANN easily falls into the local minimum and has a poor global convergence rate [20]. The further optimization of the BP–ANN has also attracted growing attention [21]. Particle swarm optimization (PSO) is an algorithm that simulates the foraging behavior of birds [22]. Khajeh and coworkers [23] integrated PSO in a BP–ANN model for the specification of optimal initial weights and threshold values by updating generations to avoid the local minimum and achieve global convergence quickly and correctly.

Establishing an efficient and reliable ANN model for predicting the behavior of electrochemical oxidation processes can reduce energy cost and is a fundamental step toward their control. The input parameters of ANN network are one of the key factors in establishing an ANN network. Oxidation–reduction potential (ORP) has been employed as an integrated indicator in various fields to describe the redox characteristic of any given chemical reaction system [24]. ORP has a good relationship with the chemical oxygen demand (COD) of electro-oxidation [25]. Wang and coworkers [26] constructed a model of the multiparameter linear relationship between ORP and Qsp (specific electrical charge) and between a COD and Cl^−1^ concentration to reflect quantitatively the effect of the current density, Cl^−1^ concentration, pollutant load, and reaction time on the electro-oxidation system. Basha et al. [27] built a BP–ANN model to predict the effect of electro-oxidation on COD removal, but ORP was not considered in the input parameters.

In this study, PSO–BP–ANN models were constructed to predict the COD removal efficiency and total energy consumption (TEC) of electro-oxidation. ORP was used as one of the input parameters. First, BP–ANN and the selection of the number of hidden layers and training algorithm were discussed in detail. Then, the PSO algorithm was used to optimize the weight and threshold of BP–ANN and identify the optimal parameters of the PSO algorithm. Experimental values were compared with output variables predicted by PSO–BP–ANN. The importance of each input variable was determined.

## 2. Materials and Methods

### 2.1. Data Set

All electro-oxidation experiments were conducted with a 3 L-capacity laboratory-scale plate cell with a circulating tank. The used datasets were obtained from a previous study [25]. A total of 190 experimental runs (Table A1) were performed in the galvanostatic mode under a current density of 8 mA cm^−2^ to 25 mA cm^−2^, an original pH of 3 to 11, an electrolyte concentration of 0.05mol L^−1^ to 0.12 mol L^−1^, a reaction time of 0 h to 2 h, and ORP values of −68 mV to 500 mV, as shown in Table 1.

During the Electro-oxidation, an ORP (SX-630, Sanxin, China) and a pH (SX711, Sanxin, China) probe were installed in the electrolysis bath for online monitoring of ORP/pH. COD was determined according to Chinese standard HJ/T 399-2007 with slight modifications. The solution was measured at a wavelength of 440 nm using a UV-visible spectrophotometer (UV-2910, Hitachi, Japan).

A specific electrical charge (*Q_sp_*, Ah L^−1^) was calculated by using the following equation [26]:

(1) $$Q_{sp}=j\cdot A\cdot \frac{t}{V}$$

where *j* is current density (A cm^−2^), *A* is the effective area of the electrode (cm^2^), *V* is the effective volume of the plate cell (L), and *t* is the reaction time during the electro-oxidation process (h).

TEC (kWh m^−3^) was calculated in a previous study as follows [28]:

(2) $$TEC=Q_{sp}\cdot U$$

where *Q_sp_* is a specific electrical charge, and *U* (V) is the cell voltage.

### 2.2. BP–ANN Coupled with PSO

**Figure 1:**
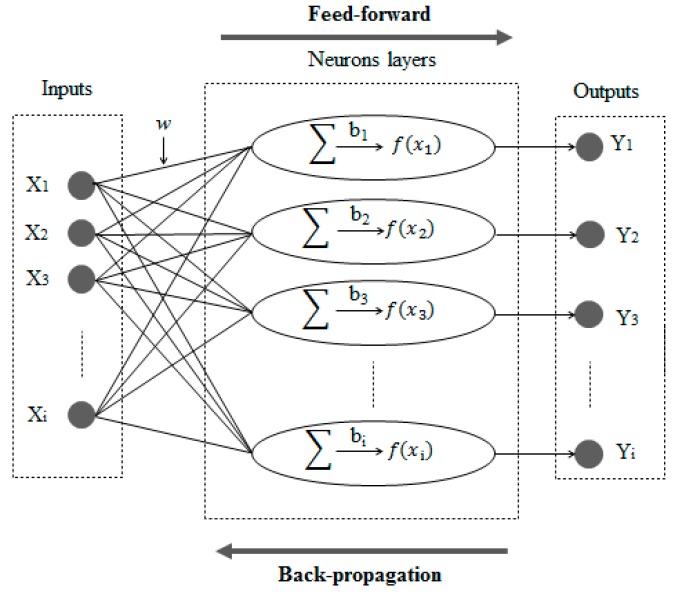
Architecture of an artificial neural network (ANN) and feed-forward back-propagation algorithm.

ANNs have different architectures. The ANN used in this study has three layers: an input layer that receives electro-oxidation information, a hidden layer that processes information, and an output layer that calculates COD removal and TEC results [29]. During BP learning, the actual outputs are compared with the target values to derive error signals, which are propagated backward by layers to adjust the weights in all lower layers [30]. The architecture of a neural network and the BP algorithm is presented in Figure 1.

**Figure 2:**
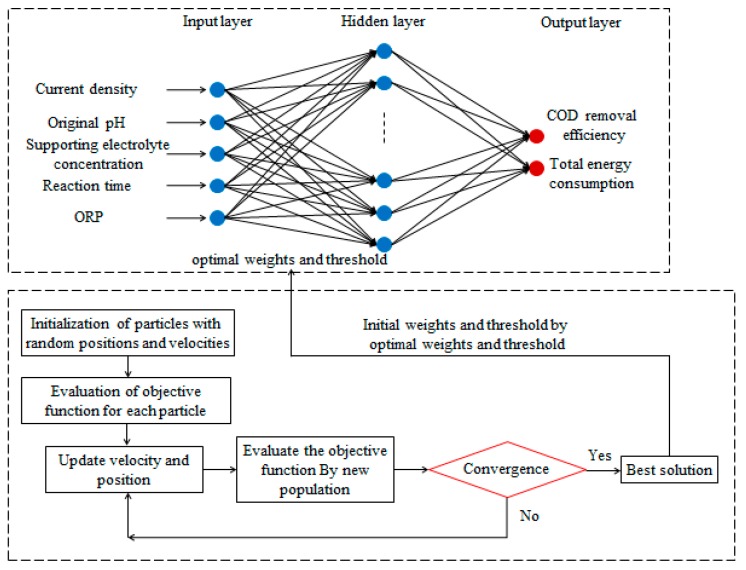
Flowchart of a backpropagation artificial neural network (BP–ANN) combined with particle swarm optimization (PSO).

The flowchart of BP–ANN coupled with PSO is shown in Figure 2. The ANN model was developed using MATLAB R2016a software. A total of 190 runs of the electro-oxidation process data were applied to develop the models for the prediction of COD removal efficiency and TEC. The available data were divided into training, validation, and testing subsets, of which 80% (152) were randomly selected for network training, 10% (19) were used for validation, and 10% (19) were applied to test network accuracy. Current density, original pH, electrolyte concentration, oxidation time, and ORP were used as five input parameters, and COD removal efficiency and TEC were considered as the two output.

Two prediction score metrics, the coefficient of correlation (R^2^), and mean square error (MSE), were computed using the following equations to evaluate the fitting and prediction accuracy of the constructed models [31]:

(3) $$R^{2}=\frac{\sum_{i=1}^{n}\left(f_{\exp,i}-F_{\exp})(f_{ANN,i}-F_{ANN}\right)}{\sqrt{\sum_{i=1}^{n}\left({\left(f_{\exp,i}-F_{\exp}\right)}^{2}{\left(f_{ANN,i}-F_{ANN}\right)}^{2}\right)}}$$

(4) $$MSE=\frac{\sum_{i=1}^{n}{\left(f_{\exp,i}-f_{ANN,i}\right)}^{2}}{n}$$

where $F_{\exp}=\frac{1}{n}\sum_{i=1}^{n}f_{\exp,i}$, $F_{ANN}=\frac{1}{n}\sum_{i=1}^{n}f_{ANN,i}$, *n* is the number of samples used for modeling, *f*_exp_ is the experimental value, and *f_ANN_* is the network-predicted value.

## 3. Results and Discussion

### 3.1. Removal Kinetics

**Figure 3:**
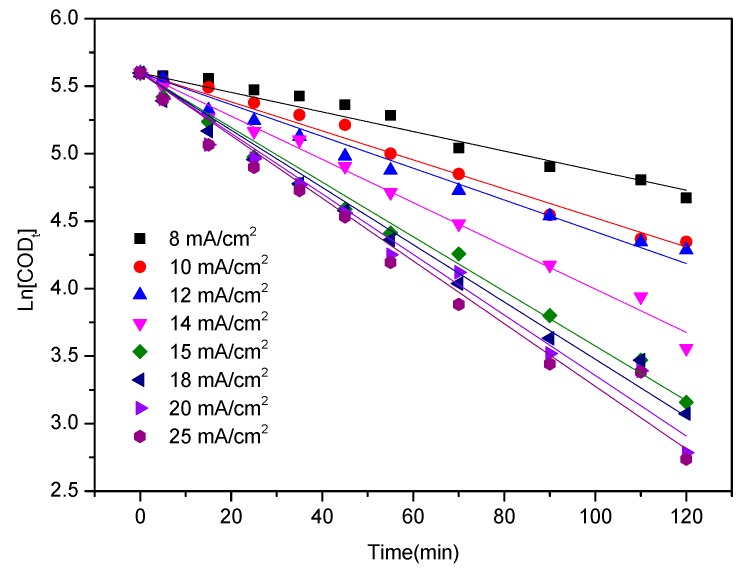
Linear relationship between the logarithmic values of chemical oxygen demand (COD) and electrolysis time.

The apparent reaction rate constants for COD removal were calculated in accordance with Equation (5) [32]:

(5) $$\ln[COD_{t}]=\ln[COD_{0}]-Kt$$

where *COD*_0_ and *COD_t_* are the COD values of the initial and final pollutant concentrations (mg L^−1^), respectively; *t* is the electrolysis time (min); and *K* is the apparent reaction rate constant (min^−1^). The apparent reaction rate constants calculated in accordance with Equation (3) for the current densities of 8, 10, 12, 14, 15, 18, 20, and 25 mA cm^−2^ were 0.0072, 0.0107, 0.0118, 0.0160, 0.0202, 0.0212, 0.0224, and 0.0232 min^−1^, respectively. The linear relationship between the logarithmic values of COD and electrolysis time is depicted in Figure 3. Table 2 shows that the correlation coefficient R^2^ of linear fitting was greater than 0.9989. This result indicates that COD removal satisfies the first-order reaction kinetics equation.

Other parameters, such as temperature (T), pH value, and electricity can be obtained when the influent quality and flow rate are held constant in the electrolytic cell. The kinetic constant K is only related to current density (*j*) under the conditions of the original pH of 3 and Na_2_SO_4_ concentration of 0.10 mol L^−1^ [11].

(6) $$K=Mj^{a}$$

The relationship between *K* and *J* can be inferred from Table 2.

(7) $$K=0.0012j^{0.9485}$$

From Equation (5), Equation (7) can be expressed as

(8) $$\ln[COD_{t}]=\ln[COD_{0}]-0.0012j^{0.9485}t$$

which describes the relationship among COD, current density, and oxidation time.

**Figure 4:**
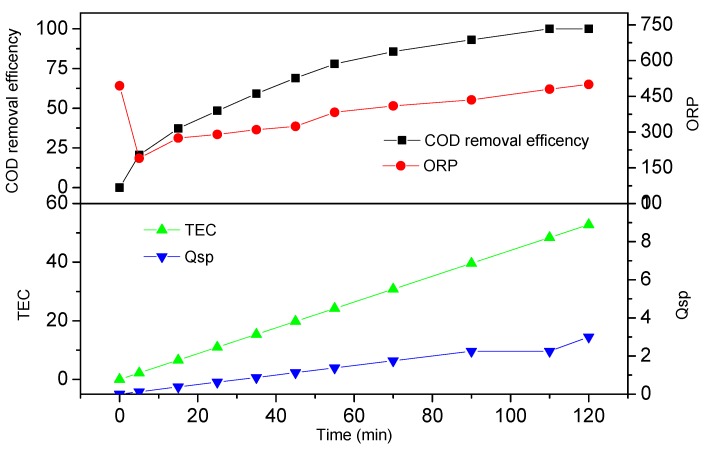
COD removal efficiency, ORP, total energy consumption (TEC), and Qsp under a current density of 15 mA cm^−2^, original pH of 3, and an Na_2_SO_4_ concentration of 0.10 mol L^−1^.

The optimal electro-oxidation conditions were initially determined by considering the effective factors of current density, original pH value, and electrolyte concentration. A COD removal efficiency of 100% was obtained with the optimal operating parameters of a current density of 15 mA cm^−2^, an original pH of 3, and a Na_2_SO_4_ concentration of 0.10 mol L^−1^ at 120 min. The dependencies of the values of COD, ORP, TEC, and Qsp under a current density of 15 mA cm^−2^, an original pH of 3, and a Na_2_SO_4_ concentration of 0.10 mol L^−1^ during electrochemical oxidation are shown in Figure 4. COD removal efficiency, TEC, and Qsp increased with electro-oxidation time. COD removal efficiency, TEC, Qsp, and ORP were 77.9%, 24.2 kWh m^−3^, 1.375 Ah L^−1^, and 383 mV, respectively, when oxidation time was 1 h. The ORP value decreased from 494 mV to 190 mV within 5 min of electrolysis and then increased gradually to 500 mV during degradation.

The typical multiple regression equation showing the relationship among ORP, current density, original pH, Na_2_SO_4_ concentration, reaction time, and COD removal efficiency was obtained as follows:

(9) $$\begin{array}{l} COD\%=-0.16276+0.00281j+0.01709pH+1.5595[Na_{2}SO_{4}]\\ +0.00495t+9.766624E-4ORP \end{array}$$

The typical multiple regression equation representing the relationship among influential parameters and TEC was obtained and is shown below:

(10) $$\begin{array}{l} TEC=-39.06431+1.97416j+0.2894pH+66.72156[Na_{2}SO_{4}]\\ +0.46082t+0.00664ORP \end{array}$$

The R^2^ values for COD removal efficiency and TEC were 0.8878 and 0.93223, respectively. These values reflect a good correlation among COD, TEC, *j*, pH, *t*, Na_2_SO_4_ concentration, and ORP. ORP values provide a complete indicator of the effect of current density, electrolyte concentration, pH, and reaction time on the performance of the electro-oxidation system. Therefore, the ORP value can be used as an effective controlling factor for the prediction of COD removal efficiency and the TEC of electro-oxidation.

### 3.2. BP–ANN Prediction of 2-Chlorophenol Removal

The tangent sigmoid was selected as the transfer function for the input layer nodes to the hidden layer, and the purelin was selected as the transfer function for the hidden layer nodes to the output layer. All data were normalized within a range of −1 and 1 before being fed to the networks to increase training speed and facilitate modeling and prediction.

**Figure 5:**
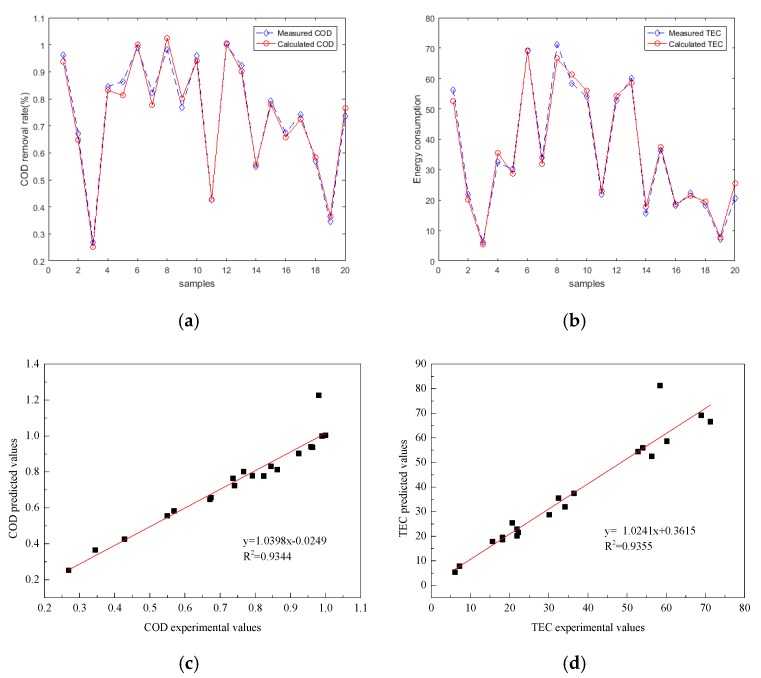
Performance of the BP–ANN predicting COD removal efficiency and TEC between experimental and predicted data sets (COD removal efficiency testing set (**a**), TEC testing set (**b**)); correlations between experimental and predicted set (COD removal efficiency testing set (**c**), TEC testing set (**d**)).

In this study, the numbers of input and output nodes were 5 and 2, respectively, and were equal to the numbers of input and output data. The number of neurons has a considerable effect on network performance. For example, the network cannot achieve the desired error if the number of neurons is too small, or overfitting may occur if the number of neurons is too large. Thus, determining the appropriate number of neurons in the hidden layer is necessary. This number can usually be determined by using the following empirical formula in accordance with Hecht–Nielsen’s theorem [33]:

(11) $$N_{H}=2N_{i}+1$$

where *N_H_* is the number of hidden neurons, and *N_i_* is the number of input variables, which is 5 in the present work. Equation (11) shows that the node number in the hidden layer was approximately 11. Then, BP networks with different hidden neurons from 6–16 were compared on the basis of the maximization of R^2^ and the minimization of MSE for the testing dataset. Table 3 shows that the BP–ANN that contains 6–16 hidden neurons in the prediction of the electro-oxidation process. The optimal BP–ANN model provided an R^2^ and MSE of 0.9344 and 0.0137232 for COD removal efficiency, respectively, and an R^2^ and MSE of 0.9355 and 0.013127 for TEC, respectively when the hidden neurons were 10. Under the optimal network, BP–ANN in the prediction of COD removal efficiency and TEC and the correlations between the experimental and predicted sets are illustrated in Figure 5. The error range of COD was (−0.058, 0.249) and TEC (−0.079, 0.391). The network performance is good, but the error range shows that the deviation of individual points is large.

The training algorithm also affects the performance of BP networks. A wide variety of training functions with 10 neurons used in the hidden layer was studied to select a good BP network. Table 4 presents the data for R^2^ and MSE under different training functions of BP networks. The Levenberg–Marquardt back propagation (trainlm) training algorithm, which maximized the R^2^ and minimized the MSE of COD removal efficiency and TEC, was identified as the best training function.

### 3.3. Optimization of the Weight and Threshold Value of BP–ANN

**Figure 6:**
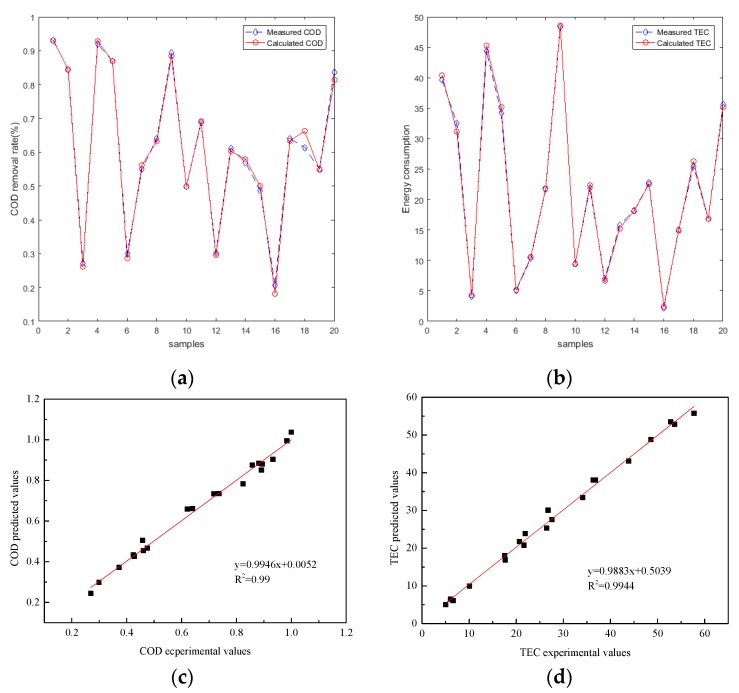
Performance of the particle swarm optimization BP–ANN (PSO–BP–ANN) predicting COD removal efficiency and TEC between experimental and predicted data sets (COD removal efficiency testing set (**a**), TEC testing set (**b**)); correlations between experimental and predicted set (COD removal efficiency testing set (**c**), TEC testing set (**d**)).

The PSO–BP–ANN can be optimized for selection purposes by optimizing (1) swarm size, (2) maximum iteration, (3) cognition coefficient C_1_, and (4) social coefficient C_2_ (Table A2). Table 5 displayed PSO control parameters, R^2^, and training MSE for the testing dataset. The PSO–ANN containing a swarm size of 50, a maximum iteration of 200, C_1_ of 1.5, and C_2_ of 1.5 was selected as the best model for the electrochemical process of interest. The optimal PSO–BP–ANN models provided R^2^ of 0.99 and 0.9944 for COD removal efficiency and TEC, and MSE values of 0.0015526 and 0.0023456, respectively, for the testing dataset. The performance of the optimal PSO–BP–ANN in the prediction of COD removal efficiency and TEC and the correlations between the experimental and predicted sets are illustrated in Figure 6. The PSO–BP–ANN selected for the efficient prediction of 2-Chlorophenol removal in an electro-oxidation system was containing 10 hidden neurons, trainlm training algorithm, swarm size of 50, maximum iteration of 200, C_1_ of 1.5, and C_2_ of 1.5.

### 3.4. Assessment of the Importance of Variables

The weight matrix of the neural net can be used to assess the relative importance of various input variables for output variables [31]. The relative importance of input variables on the value of COD removal efficiency and TEC as calculated by particle swarm optimization BP–ANN (PSO–BP–ANN) is shown in Table 6. Sensitivity analysis indicated order of relative importance the operational parameters on the electro-oxidation as: electrolysis time > pH > electrolyte concentration > ORP > current density. The table indicates that all of the variables have strong effects on COD removal efficiency and TEC. Therefore, none of the variables studied in this work should be neglected in the analysis.

## 4. Conclusions

In this study, the main object is development and construction of novel model that could make efficient prediction of electro-oxidation removal of 2-Chlorophenol on the basis of batch electro-oxidation experiments. The analysis of removal kinetics indicated that ORP was closely correlated with COD removal efficiency and TEC and was one of the important input parameters of PSO–BP–ANN. PSO–BP–ANN was developed through the optimization of the weights and thresholds of BP–ANN. The PSO–BP–ANN that contained 10 hidden neurons, trainlm training algorithm and possessed a swarm size of 50, maximum iteration of 200, C_1_ of 1.5, and C_2_ of 1.5 was identified as the best model for predicting 2-chlorophenol degradation through electro-oxidation. The PSO–BP–ANN model provided accurate predictions and R^2^ of 0.99 and 0.9944 for COD removal efficiency and TEC, and MSE values of 0.0015526 and 0.0023456 respectively for the testing dataset. The weight matrix revealed that the order of relative importance for the operational parameters of the electro-oxidation is: electrolysis time > pH > electrolyte concentration > ORP > current density. For comparative purposes, performance data for the ANN methodology in various electrochemical processes are summarized in Table A3.

## Figures and Tables

**Table 1:** Experimental conditions. ORP, oxidation–reduction potential.

Run no.	Current Density (mA cm^−2^)	Na_2_SO_4_ Concentration (mol L^−1^)	Initial pH	Electrolysis Time (h)	ORP	Flow Mode
0–190	8–25	0.05–0.12	3–11	0–2	−68–500	continuous

**Table 2:** K and correlation coefficient values under various current densities.

Current Density *j* (mA cm^−2^)	Regression Line	K (min^−1^)	R^2^
8	Y = 0.00724x + 5.59842	0.0072	0.9999
10	Y = −0.01074x + 5.59842	0.0107	0.9999
12	Y = −0.01177x + 5.59842	0.0118	0.9998
14	Y = −0.01602x + 5.59842	0.0160	0.9998
15	Y = −0.02023x + 5.59842	0.0202	0.9997
18	Y = −0.02121x + 5.59842	0.0212	0.9995
20	Y = −0.02242 + 5.59842	0.0224	0.9992
25	Y = −0.02322 + 5.59842	0.0232	0.9989

**Table 3:** Evaluation of the prediction performance of the BP–ANN model for the testing dataset.

*N_H_*	COD Removal Efficiency		TEC	
	R^2^	MSE	R^2^	MSE
6	0.9151	0.0155151	0.9277	0.014145
7	0.8741	0.0127321	0.8896	0.013234
8	0.8781	0.0152728	0.9025	0.016566
9	0.9292	0.0149617	0.9148	0.003826
10	0.9344	0.0137232	0.9355	0.013127
11	0.8998	0.0146919	0.9051	0.016887
12	0.8447	0.0165818	0.9077	0.014058
13	0.9032	0.0141709	0.9185	0.013157
14	0.8231	0.0158827	0.893	0.016551
15	0.874	0.0165818	0.8987	0.014344
16	0.8451	0.0153163	0.9021	0.013923

**Table 4:** Predictions of backpropagation (BP) models with different training algorithms for the testing dataset.

BP–ANN	Training Function	COD Removal Efficiency		TEC	
		R^2^	MSE	R^2^	MSE
Batch training with weight and bias learning rules	trainb	0.86209	0.0134868	0.88977	0.0162386
BFGS quasi-Newton backpropagation	trainbfg	0.90721	0.0161285	0.77684	0.0184532
Bayesian regularization backpropagation	trainbr	0.8426	0.012	0.84645	0.0157329
Unsupervised batch training with weight and bias learning rules	trainbu	0.91427	0.0143475	0.84693	0.0159821
Cyclical order weight/bias training	trainc	0.79387	0.0183421	0.78352	0.0173493
Powell-Beale conjugate gradient backpropagation	traincgb	0.84096	0.0183258	0.81842	0.016399
Fletcher-Reeves conjugate gradient backpropagation	traincgf	0.88913	0.0159525	0.89006	0.0144586
Polak-Ribi’ere conjugate gradient backpropagation	traincgp	0.89724	0.0153866	0.73305	0.0191479
Batch gradient descent	traingd	0.91312	0.016002	0.88845	0.0158414
Gradient descent with adaptive learning rate back propagation	traingda	0.91939	0.0191324	0.88416	0.0159636
Batch gradient descent with momentum	traingdm	0.88482	0.0163147	0.85786	0.0184368
Variable learning rate backpropagation	traingdx	0.91799	0.0143824	0.78431	0.0189369
Levenberg–Marquardt back-propagation	trainlm	0.9344	0.0137232	0.9355	0.013127

**Table 5:** PSO–ANN with different parameters of the PSO algorithm.

Number of Neurons	Swarm Size	Max Iteration	Cognition Coefficient (C_1_)	Social Coefficient (C_2_)	COD Removal Efficiency		TEC	
					R^2^	MSE	R^2^	MSE
10	10	200	1.5	1.5	0.9528	0.0024367	0.9781	0.0024975
10	30	200	1.5	1.5	0.9783	0.0034865	0.9878	0.0022
10	50	200	1.5	1.5	0.99	0.0015526	0.9944	0.0023456
10	70	200	1.5	1.5	0.976	0.0015874	0.9878	0.0038921
10	100	200	1.5	1.5	0.9736	0.00173	0.9977	0.003281
10	120	200	1.5	1.5	0.98	0.0019062	0.9983	0.0031672
10	50	100	1.5	1.5	0.9852	0.0011566	0.9834	0.0012677
10	50	150	1.5	1.5	0.9695	0.0021488	0.9876	0.001835
10	50	250	1.5	1.5	0.9891	0.0012508	0.9812	0.0033047
10	50	200	0.5	2.5	0.9767	0.0024646	0.9882	0.0026686
10	50	200	1	2	0.9888	0.00179873	0.9891	0.0012586
10	50	200	2	1	0.9874	0.0023016	0.9919	0.0034017

**Table 6:** Relative importance of input variables on the value of COD removal efficiency and TEC.

Input Variable	Importance (%)
current density	18.85%
original pH	21.11%
electrolyte concentration	19.69%
electro-oxidation time	21.30%
ORP	19.05%
Total	100%

## Appendix A

**Table A1:** The results of the electro-oxidation experiment.

Number	Current Density	pH	Na_2_SO_4_ Concentration	Time	ORP	COD Removal Efficiency	TEC
1	8	6.5	0.1	5	12	0.092	0.940
2	10	6.5	0.1	5	14	0.112	1.354
3	14	6.5	0.1	5	71	0.117	2.018
4	15	6.5	0.1	5	80	0.123	2.250
5	16	6.5	0.1	5	125	0.120	2.443
6	18	6.5	0.1	5	130	0.136	3.128
7	20	6.5	0.1	5	144	0.143	3.133
8	25	6.5	0.1	5	150	0.160	3.958
9	15	3	0.1	5	190	0.206	2.200
10	15	4	0.1	5	180	0.183	2.406
11	15	5	0.1	5	173	0.163	2.313
12	15	7	0.1	5	60	0.151	2.506
13	15	9	0.1	5	-38	0.119	2.525
14	15	11	0.1	5	-68	0.088	2.434
15	15	3	0.05	5	162	0.105	2.025
16	15	3	0.08	5	183	0.153	2.438
17	15	3	0.1	5	190	0.206	2.200
18	15	3	0.12	5	180	0.183	2.688
19	8	6.5	0.1	15	50	0.231	2.820
20	10	6.5	0.1	15	61	0.271	4.063
21	12	6.5	0.1	15	73	0.299	5.019
22	14	6.5	0.1	15	100	0.305	6.055
23	15	6.5	0.1	15	140	0.322	6.750
24	16	6.5	0.1	15	190	0.332	7.328
25	20	6.5	0.1	15	220	0.372	9.400
26	25	6.5	0.1	15	230	0.423	11.875
27	15	3	0.1	15	275	0.372	6.600
28	15	4	0.1	15	210	0.345	7.219
29	15	5	0.1	15	187	0.302	6.938
30	15	7	0.1	15	120	0.287	7.519
31	15	9	0.1	15	56	0.248	7.575
32	15	11	0.1	15	13	0.195	7.301
33	15	3	0.05	15	250	0.269	6.075
34	15	3	0.08	15	265	0.324	7.313
35	15	3	0.1	15	275	0.372	6.600
36	15	3	0.12	15	230	0.360	8.063
37	8	6.5	0.1	25	60	0.338	4.700
38	10	6.5	0.1	25	70	0.394	6.771
39	12	6.5	0.1	25	80	0.438	8.365
40	14	6.5	0.1	25	112	0.461	10.092
41	15	6.5	0.1	25	156	0.484	11.250
42	16	6.5	0.1	25	224	0.499	12.213
43	18	6.5	0.1	25	256	0.544	15.638
44	20	6.5	0.1	25	259	0.550	15.667
45	25	6.5	0.1	25	270	0.623	19.792
46	15	3	0.1	25	290	0.484	11.000
47	15	4	0.1	25	231	0.450	12.031
48	15	5	0.1	25	201	0.396	11.563
49	15	7	0.1	25	145	0.377	12.531
50	15	9	0.1	25	85	0.349	12.625
51	15	11	0.1	25	44	0.291	12.169
52	15	3	0.05	25	278	0.384	10.125
53	15	3	0.08	25	280	0.456	12.188
54	15	3	0.1	25	290	0.484	11.000
55	15	3	0.12	25	245	0.493	13.438
56	8	6.5	0.1	35	73	0.428	6.580
57	10	6.5	0.1	35	80	0.498	9.479
58	12	6.5	0.1	35	91	0.556	11.711
59	14	6.5	0.1	35	125	0.584	14.128
60	15	6.5	0.1	35	170	0.612	15.750
61	16	6.5	0.1	35	240	0.637	17.099
62	18	6.5	0.1	35	260	0.671	21.893
63	20	6.5	0.1	35	273	0.688	21.933
64	25	6.5	0.1	35	283	0.752	27.708
65	15	3	0.1	35	310	0.592	15.400
66	15	4	0.1	35	240	0.550	16.844
67	15	5	0.1	35	210	0.472	16.188
68	15	7	0.1	35	180	0.458	17.544
69	15	9	0.1	35	100	0.424	17.675
70	15	11	0.1	35	53	0.363	17.036
71	15	3	0.05	35	292	0.477	14.175
72	15	3	0.08	35	305	0.566	17.063
73	15	3	0.1	35	310	0.592	15.400
74	15	3	0.12	35	303	0.588	18.813
75	8	6.5	0.1	45	85	0.497	8.460
76	10	6.5	0.1	45	110	0.574	12.188
77	12	6.5	0.1	45	115	0.640	15.057
78	14	6.5	0.1	45	153	0.674	18.165
79	15	6.5	0.1	45	172	0.706	20.250
80	16	6.5	0.1	45	248	0.736	21.984
81	18	6.5	0.1	45	270	0.762	28.148
82	20	6.5	0.1	45	287	0.786	28.200
83	25	6.5	0.1	45	292	0.836	35.625
84	15	3	0.1	45	324	0.690	19.800
85	15	4	0.1	45	260	0.640	21.656
86	15	5	0.1	45	218	0.546	20.813
87	15	7	0.1	45	190	0.543	22.556
88	15	9	0.1	45	101	0.487	22.725
89	15	11	0.1	45	66	0.428	21.904
90	15	3	0.05	45	301	0.569	18.225
91	15	3	0.08	45	318	0.656	21.938
92	15	3	0.1	45	324	0.690	19.800
93	15	3	0.12	45	310	0.700	24.188
94	8	6.5	0.1	55	90	0.550	10.340
95	10	6.5	0.1	55	120	0.640	14.896
96	12	6.5	0.1	55	130	0.704	18.403
97	14	6.5	0.1	55	170	0.741	22.202
98	15	6.5	0.1	55	179	0.774	24.750
99	16	6.5	0.1	55	250	0.804	26.869
100	18	6.5	0.1	55	276	0.819	30.525
101	20	6.5	0.1	55	286	0.840	34.467
102	25	6.5	0.1	55	293	0.884	43.542
103	15	3	0.1	55	383	0.779	24.200
104	15	4	0.1	55	288	0.718	26.469
105	15	5	0.1	55	256	0.614	25.438
106	15	7	0.1	55	205	0.621	27.569
107	15	9	0.1	55	106	0.545	27.775
108	15	11	0.1	55	83	0.475	26.771
109	15	3	0.05	55	312	0.651	22.275
110	15	3	0.08	55	353	0.734	26.813
111	15	3	0.1	55	383	0.779	24.200
112	15	3	0.12	55	363	0.765	29.563
113	8	6.5	0.1	70	97	0.617	13.160
114	10	6.5	0.1	70	124	0.717	18.958
115	12	6.5	0.1	70	160	0.782	23.422
116	14	6.5	0.1	70	186	0.819	28.257
117	15	6.5	0.1	70	193	0.849	31.500
118	16	6.5	0.1	70	251	0.869	34.197
119	18	6.5	0.1	70	282	0.891	43.785
120	20	6.5	0.1	70	288	0.902	43.867
121	25	6.5	0.1	70	292	0.925	55.417
122	15	3	0.1	70	410	0.857	30.800
123	15	4	0.1	70	305	0.804	33.688
124	15	5	0.1	70	280	0.712	32.375
125	15	7	0.1	70	215	0.728	35.088
126	15	9	0.1	70	127	0.635	35.350
127	15	11	0.1	70	87	0.557	34.073
128	15	3	0.05	70	321	0.721	28.350
129	15	3	0.08	70	383	0.824	34.125
130	15	3	0.1	70	410	0.857	30.800
131	15	3	0.12	70	380	0.826	37.625
132	8	6.5	0.1	90	105	0.691	16.920
133	10	6.5	0.1	90	139	0.791	24.375
134	12	6.5	0.1	90	160	0.863	30.114
135	14	6.5	0.1	90	198	0.882	36.330
136	15	6.5	0.1	90	205	0.914	40.500
137	16	6.5	0.1	90	252	0.924	43.968
138	18	6.5	0.1	90	282	0.963	56.295
139	20	6.5	0.1	90	291	0.975	56.400
140	25	6.5	0.1	90	296	0.981	71.250
141	15	3	0.1	90	435	0.931	39.600
142	15	4	0.1	90	356	0.89	43.313
143	15	5	0.1	90	313	0.834	41.625
144	15	7	0.1	90	230	0.813	45.113
145	15	9	0.1	90	146	0.729	45.450
146	15	11	0.1	90	98	0.663	43.808
147	15	3	0.05	90	335	0.792	36.450
148	15	3	0.1	90	435	0.931	39.600
149	15	3	0.12	90	423	0.893	48.375
150	8	6.5	0.1	110	121	0.737	20.680
151	10	6.5	0.1	110	140	0.827	29.792
152	12	6.5	0.1	110	173	0.894	36.806
153	14	6.5	0.1	110	193	0.920	44.403
154	15	6.5	0.1	110	210	0.947	49.500
155	16	6.5	0.1	110	254	0.951	53.739
156	18	6.5	0.1	110	285	0.981	68.805
157	20	6.5	0.1	110	291	0.990	68.933
158	25	6.5	0.1	110	293	1.000	87.083
159	15	3	0.1	110	480	1.000	48.400
160	15	5	0.1	110	330	0.931	50.875
161	15	7	0.1	110	235	0.900	55.138
162	15	9	0.1	110	150	0.809	55.550
163	15	11	0.1	110	108	0.740	53.543
164	15	3	0.05	110	367	0.846	44.550
165	15	3	0.08	110	412	0.933	53.625
166	15	3	0.1	110	480	1	48.400
167	15	3	0.12	110	430	0.927	59.125
168	8	6.5	0.1	120	125	0.76	22.560
169	10	6.5	0.1	120	143	0.845	32.500
170	12	6.5	0.1	120	182	0.911	40.152
171	14	6.5	0.1	120	189	0.933	48.440
172	15	6.5	0.1	120	215	0.959	54.000
173	16	6.5	0.1	120	256	0.96	58.624
174	18	6.5	0.1	120	283	0.99	75.060
175	20	6.5	0.1	120	291	1	75.200
176	25	6.5	0.1	120	292	1	95.000
177	15	3	0.1	120	500	1	52.800
178	15	4	0.1	120	420	0.984	57.750
179	15	5	0.1	120	346	0.953	55.500
180	15	7	0.1	120	240	0.924	60.150
181	15	9	0.1	120	152	0.832	60.600
182	15	11	0.1	120	115	0.767	58.410
183	15	3	0.05	120	370	0.858	48.600
184	15	3	0.08	120	435	0.936	58.500
185	15	3	0.1	120	500	1.000	52.800
186	15	3	0.12	120	435	0.940	64.500
187	12	6.5	0.1	5	43	0.118	1.673
188	18	6.5	0.1	15	200	0.37	9.383
189	15	4	0.1	110	380	0.957	52.938
190	15	3	0.08	90	400	0.891	43.875

**Table A2:** Partly of PSO–BP–ANN training function code.

Training Function Code
net = newff(inputn,outputn,hiddennum,{‘logsig’,‘purelin’},‘traingdx’); c1 = 1.5; c2 = 1.5; maxgen = 200; sizepop = 50; Vmax = 1; Vmin = −1; popmax = 5; popmin = −5; for *i* = 1:sizepop pop(*i*,:) = 5 * rands(1,numsum); V(*i*,:) = 1 * rands(1,numsum); fitness(*i*) = fun(pop(*i*,:),inputnum,hiddennum,outputnum,net,inputn,outputn); [bestfitness bestindex] = min(fitness); zbest = pop(bestindex,:); gbest = pop; fitnessgbest = fitness; fitnesszbest = bestfitness; for *i* = 1:maxgen V(*j*,:) = w * V(*j*,:) + c1 * rand * (gbest(*j*,:) − pop(*j*,:)) + c2 * rand * (zbest − pop(*j*,:)); V(*j*,find(V(*j*,:) > Vmax)) = Vmax; V(*j*,find(V(*j*,:) < Vmin)) = Vmin; pop(*j*,:) = pop(*j*,:) + 0.2 * V(*j*,:); pop(*j*,find(pop(*j*,:) > popmax)) = popmax; pop(*j*,find(pop(*j*,:) < popmin)) = popmin; pos = unidrnd(numsum); if rand > 0.95 pop(j,pos) = 5 * rands(1,1); fitness(j) = fun(pop(j,:),inputnum,hiddennum,outputnum,net,inputn,outputn); for *j* = 1:sizepop if fitness(*j*) < fitnessgbest(*j*) gbest(*j*,:) = pop(*j*,:); fitnessgbest(*j*) = fitness(*j*); if fitness(*j*) < fitnesszbest zbest = pop(*j*,:); fitnesszbest = fitness(*j*);

**Table A3:** ANN models for applications in various electrochemical processes.

Type of Process	Input Variable	Output Variable	Types of the ANN Model	R^2^	References
electrocoagulation	Current density, electrolysis time, initial pH and dye concentration, conductivity, retention time of sludge and distance between electrodes	Color removal efficiency	BP–ANN	0.974	Daneshvar et al. [16]
electro-oxidation	Intensity of current, reaction time, pH, nature of electrolyte, concentration of electrolyte	Degradation rate of oxytetracycline	BP–ANN	0.99	Belkacem et al. [19]
electrochemically activated persulfate	Electrolysis time, applied current, persulfate, pH	Sulfamethoxazoleremoval efficicency	BP–ANN	0.9398	Zhang et al. [10]
electrocoagulation-flotation	Initial HA concentration, initial pH, electrical conductivity, current density, number of pulses	Humica acid	BP–ANN	0.966	Hasani et al. [34]
